# Global health actors no longer in favor of user fees: a documentary study

**DOI:** 10.1186/1744-8603-9-29

**Published:** 2013-07-26

**Authors:** Emilie Robert, Valéry Ridde

**Affiliations:** 1 Centre de recherche du Centre hospitalier de l’Université de Montréal (CRCHUM), Faculté de médecine, Université de Montréal, Pavillon Masson, 3850, rue Saint-Urbain, Montréal (Québec) H2W 1T7C, CANADA; 2 Département de médecine sociale et préventive, Université de Montréal, Québec, Canada

**Keywords:** User fees, LMICs, International health policy, Global health actors, Policy change

## Abstract

**Background:**

Since the advent of health user fees in low- and middle-income countries in the 1980s, the discourse of global health actors (GHAs) has changed to the disadvantage of this type of healthcare financing mechanism. The aim of the study was to identify and analyze the stance of GHAs in the debate on user fees.

**Methods:**

We conducted documentary research using public documents published by and officially attributed to GHAs from 2005 to 2011. We categorized GHAs into four groups: intergovernmental organizations, international non-governmental organizations, government agencies, and working groups and networks. We then classified the GHAs according to their stance relative to the abolition of user fees, and conducted a thematic analysis of their discourse to understand the arguments used by each GHA to justify its stance.

**Results:**

We identified 56 GHAs, for which we analyzed 140 documents. Among them, 55% were in favor of the abolition of user fees or in favor of free care at the point of delivery. None of the GHAs stated that they were in favor of user fees; however, 30% did not take a stand. Only the World Bank declares that it is both in favor of user fees and in favor of free care at point of service. GHAs generally circumscribe their stance to specific populations (pregnant women, children under 5 years, etc.) or to specific health services (primary, basic, essential). Three types of arguments are used by GHAs to justify their stance: economic, moral and ethical, and pragmatic.

**Conclusions:**

The principle of “user pays” seems to have fizzled. Production and dissemination of evidence, as well as certain advocacy networks, may have contributed to this change in discourse. However, GHAs should go a step further and translate their words into action, so that free healthcare at the point of delivery becomes a reality in low- and middle-income countries. They should provide technical and financial support to those countries that have chosen to implement user fee exemption policies, sometimes influenced by a GHA.

## Abstract (fr)

**Résumé:**

**Contexte**

Depuis l′avènement des paiements directs des soins dans les années 1980 dans les pays à faible et moyen revenu, le discours des acteurs de la santé mondiale a évolué en défaveur de ce mode de financement de la santé. L’objectif de l’étude est de déterminer et d’analyser la position des acteurs de la santé mondiale dans le débat sur les paiements directs.

**Méthodes**

Nous avons mené une recherche documentaire à partir de l’analyse des documents publiés et officiellement attribués aux acteurs de la santé mondiale entre 2005 et 2011. Nous avons catégorisé les acteurs en quatre groupes : les organisations intergouvernementales, les organisations internationales non gouvernementales, les agences gouvernementales de coopération, et les réseaux et groupes de travail transnationaux. Nous avons ensuite classé chaque acteur selon sa position par rapport à l’abolition des paiements directs. Nous avons enfin mené une analyse thématique des discours pour comprendre les arguments utilisés par chaque acteur pour défendre sa position.

**Résultats**

Nous avons répertorié 56 acteurs de la santé mondiale pour lesquels nous avons retenu 140 documents. Parmi eux, 55% sont en faveur de l’abolition des paiements directs des soins ou de la gratuité des soins au point de service. Si aucun acteur ne se prononce plus en faveur des paiements directs, 30% ne prennent pas position. Seule la Banque Mondiale s'exprime à la fois en faveur des paiements directs et en faveur de la gratuité des soins au point de service. Les acteurs circonscrivent généralement leur position positive à certaines populations (femmes enceintes, enfants de moins de 5 ans, etc.) ou à certains soins (soins primaires, de base, essentiels). Trois types d’arguments sont utilisés par les acteurs pour défendre leur position : économique, moral/éthique et pragmatique.

Le principe de l’utilisateur-payeur semble avoir fait long feu. Les données scientifiques et certains réseaux d’acteurs ont sans doute contribué à ce changement de discours. Pour autant, les acteurs de la santé mondiale qui ont maintenant trouvé un consensus ne doivent plus se contenter de prendre position. Ils doivent transformer leurs paroles en actes et soutenir tant techniquement que financièrement les pays qui ont choisi de mettre en place des mesures d’exemption, parfois sous leur influence.

## Background

Universal health coverage is the new objective of the decade [1] and several low-and middle-income countries (LMICs), such as India and Ghana, are now testing the principle of sharing the financial risks linked with access to healthcare. However, such policies are few in number, especially in Africa [2], and user fees remain a widespread health financing mechanism [3,4].

The issue of health user fees has been fiercely debated [5] since the publication of the De Ferranti report [6], and the Agenda for Reform by the World Bank [7], and the implementation of the Bamako Initiative in the late 1980s. The financial participation of users of health services, by means of user fees, was regarded as a way to improve community participation and access to primary health care [8], where health systems were weak and underfunded. Many African countries (e.g. Burkina Faso, Niger, Senegal, South Africa, Uganda, etc.) and some countries in Asia (e.g. Burma, Vietnam, Cambodia) and in Latin America (e.g. Peru, Honduras) reformed their health system based on this principle [9].

Even in the early years of implementing the Bamako Initiative in Africa and Asia, voices were raised to denounce the potential risks of such an approach for the most disadvantaged populations. As a case in point, the Canadian Public Health Association declared in 1990: “*The financial requirements of attaining and sustaining PHC* [primary health care] *must not be met by imposing an increased financial burden on the poorest and most vulnerable in society* […]” [10]. Studies in LMICs [11,12] have regularly shown that user fees deterred the use of health services and threatened equity [13,14]. However, the evidence is mixed [11,15], showing, for example, that quality of care improved in some settings.

Currently there seems to be few strong supporters of user fees. Indeed, in a context where the objectives of the Millennium Development Goals are still far from being achieved, particularly in African countries [16], health user fees are major barriers to access to care for vulnerable populations [17]. More than fifteen years have passed since South Africa has decided to make health care free for a certain part of its population in 1994. This country paved the way for its neighbors. Today, despite the multiple setbacks faced by user fee exemption policies, specifically with regards to implementation [18], around twenty LMICs, supported by several international donors, have followed the example of South Africa [19].

Some feel that there is now a consensus on the need to abolish user fees [20,21]: “*A consensus is emerging that user fees are not an appropriate financing mechanism for health services in developing countries*” [20]. The Lancet editorial of September 2012 with its categorical statement that direct payments “*should be scrapped*” [22] seems to confirm this growing opinion on the global health scene.

In this study, we sought to determine to what extent a consensus exists among the various global health actors (GHAs) on the issue of user fees in LMICs. The objective is therefore to identify and analyze the public position statements of GHAs in the debate.

## Methods

### Research design

As the example of the Bamako Initiative shows, more and more health reforms and public health policies are initiated at the supranational level. The “*global agora*” [23] is now the place where public policy processes, notably agenda setting, are taking place. Multiple actors, and not only states, are involved in priority setting in LMICs [24]. Moreover, the debate on user fees far exceeds the frontiers of the countries in which they are implemented. We thus decided to focus on the supranational debate involving GHAs, rather than local or state actors.

In the frame of a study on a supranational debate, collecting primary data from stakeholders is hardly feasible. It is therefore possible to base the study on secondary data, as explained by Varvasovsky and Brugha [25] : “*A supra*-*national analysis*, *involving international actors*, *is likely to rely more on a review of policy documents*, *reports and existing data*” (p.340). We therefore conducted documentary research, facilitated by the abundance of documents published by GHAs and their accessibility. This type of study also has the advantage of limiting the influence of the researcher on the study material [26].

### Identification of stakeholders

According to Varvasovsky and Brugha [25], stakeholders are “*actors who have an interest in the issue under consideration*, *who are affected by the issue*, *or who* – *because of their position* – *have or could have an active or passive influence on the decision*-*making process*”.

We initially classified the different types of GHAs according to the categorization proposed by Evans and colleagues [27]. They distinguish between: 1) intergovernmental organizations (IGOs), composed mainly of member states and established by treaty, giving them a legal international status, and 2) international non-governmental organizations (INGOs), which are voluntary associations composed of individuals or organizations for international action. In order to take into consideration the new GHAs [28], we also included: 3) government agencies (GAs), national development aid agencies that act in the frame of bilateral agreements, mostly involving high-income countries; and 4) working groups and transnational networks (NW/WGs). Indeed, the globalization of trade is accompanied by the emergence of a new category of actors whose voice on the global health scene is gradually growing. Heterogeneous by nature, these actors can be either formal working groups formed by international experts or representatives of organizations for a specific purpose or mission (e.g. The Task Force on Global Action for Health System Strengthening, called also the "Takemi Working Group") or transnational networks that are formed from a common interest (e.g. MDG Africa Steering Group, a group of leader intergovernmental organizations). These actors are developing rapidly and produce many reports that are widely disseminated.

For each category, a list of major GHAs was compiled through an iterative process (Table 1). According to our knowledge of the context, we initially identified GHAs according to their involvement – at the global level – in health care financing and/or access to health care discussions. We then used the snowballing technique to complete this list: we added to the list any new GHA mentioned by previously identified GHAs and who met the two criteria mentioned above. An expert in global health also recommended the inclusion of other GHAs.

**Table 1:** Categorization of GHAs

**Intergovernmental organizations (IGOs)**	**Governments and their agencies (GAs)**	**International non-governmental organizations (INGOs)**	**Transnational networks, task forces and working groups**		
			**Networks of INGOs**	**Networks of GAs and IGOs**	**Other**
African Development Bank*	Germany (GIZ)	Bill and Melinda Gates Foundation	Action for Global Health	Commission for Africa	Countdown to 2015
Asian Development Bank	Canada (CIDA)	Médecins du Monde	Action mondiale contre la pauvreté	G8	Global Health Council
African Union	Denmark (DANIDA)	Médecins Sans Frontière	Coordination Sud	MDG Africa Steering Group	International Health Partnership + *
Council of the European Union	United States (USAID)	Merlin	Global Health Watch	The Global Campaign for health MDGs	People’s Health Movement
European Commission	Japan (JICA)	Rockefeller Foundation	The Global Call to Action Against Poverty		
European Parliament	France (AFD)	Oxfam	InterAction	UNAIDS	Task Force on Global Action for Health System Strengthening
					Taskforce on innovative international financing for health systems
					The Global Coalition on Women and AIDS
					UN Millennium Project
					P4H
Humanitarian Aid Department of the European Commission (ECHO)	Netherlands	Save the Children			
Inter-American Development Bank	Norway	World Vision			
International Labour Organization (ILO)	Sweden				
Organization for Economic Co-operation and Development (OECD)	United Kingdom (DFID)				
The World Bank					
United Nations Children's Fund (UNICEF)					
United Nations Development Programme (UNDP)					
United Nations General Assembly					
United Nations Population Fund (UNFPA)					
West African Health Organization (WAHO)					
World Health Assembly					
World Health Organization (WHO)					
			**n = 6**	**n = 5**	**n = 9**
**n = 18**	**n = 10**	**n = 8**	**n = 20**		

* *No document was found for these GHAs*.

### Data collection

Insofar as we are interested in a supranational debate in the public arena, we chose to only analyze the official and public position statements of GHAs. Because the World Wide Web has become the major public relations medium [29], we posited that GHAs willing to express a public statement would use this channel of information to inform the public and spread their message. As a consequence, we opted to look for documents available on the Internet. We also considered that documents that are not public or that are under disclosure should not be part of our analysis because they are not accessible to the public. Furthermore, because we focused on the Internet as the only source of information, we ensured that the process of data collection was as transparent, systematic and unbiased as possible.

The official websites of the listed GHAs were systematically and comprehensively consulted to find any public documents. To be included in the study, a document had to: 1) be in English or in French; 2) be published between 2005 and 2011; 3) be published by a GHA; 4) address the issue of development, poverty or healthcare in LMICs; 5) be officially credited by the GHA (documents marked “*the views expressed in this paper are solely those of the author*” and similar were excluded); 6) present a point of view, a strategy, a position, a call, a plan of action, and not just an activity report.

In order to get the most accurate vision of the possible positions of the GHAs, the "snowballing" technique was applied to selected documents. This technique allowed us to take into account new actors that were gradually added to the list of stakeholders. Key informants, whose research interests focus on the issues of health system financing in LMICs, access to health care for vulnerable populations and equity, were also contacted. These persons directed us to available documentation from conferences or international meetings.

### Data analysis

As a first step, we mapped the positions of the GHAs in the debate on the abolition of user fees. We took the features of the stakeholders’ analysis, which is used to understand the role of stakeholders in the political decision-making process. This provides a focus on policy stakeholders, their interests and their relationships [25], notably on health policy issues [30]. Such studies were recently conducted in LMICs about universal health coverage in Tanzania [31] and about the influence of research on health systems in the policy-making process [32].

**Figure 1:**
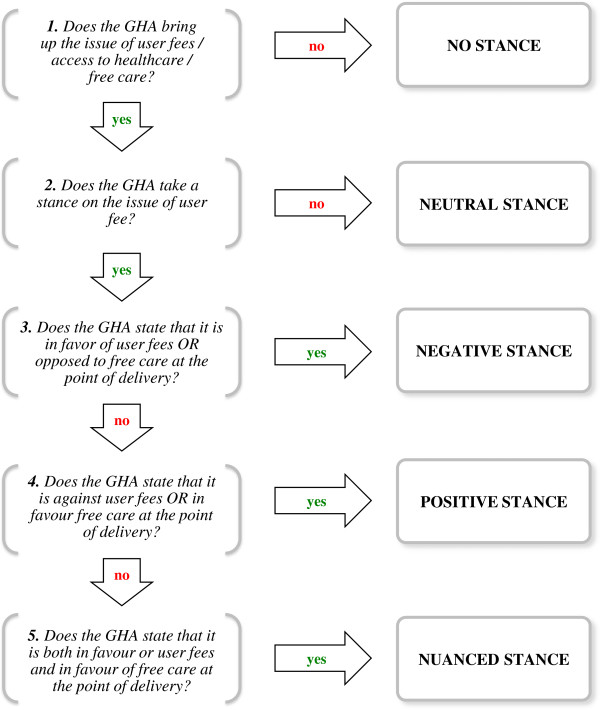
Decision tree to classify GHAs according to their stance on the issue of user fees.

A decision tree (Figure 1) guided the classification of the GHAs according to their position on the issue of user fees. It classifies the GHAs into five groups: no stance, neutral stance, negative stance, positive stance and nuanced stance. When multiple documents were assigned to one GHA, the most recent document in which a position was identified was used to determine the GHA’ s position.

For each document, passages that were relevant to the study were transcribed into a Microsoft Excel matrix for easier data handling. The lead author applied the decision tree to all passages. A research assistant applied the item 2 “*Does the GHA take a stance on the issue of user fees*?” to all passages, except those already categorized “*No stance*”. In order to limit observer bias, this step was carried out blinded to knowledge of the authors of the passages. An inter-observer agreement was calculated for item 2 using Cohen's kappa coefficient [33]. The coefficient obtained was 88.9%. Disagreements were resolved by consensus.

As a second step, the lead author conducted a thematic analysis of the GHAs’ discourse to identify what arguments were used to justify their position. Mapping the positions of the GHAs allowed us to inductively develop a coding grid. This grid was applied to the passages compiled in the Microsoft Excel matrix, or to the entire document when necessary. It was iteratively completed before being finalized.

## Results

### Description of the GHAs and included documents

**Figure 2:**
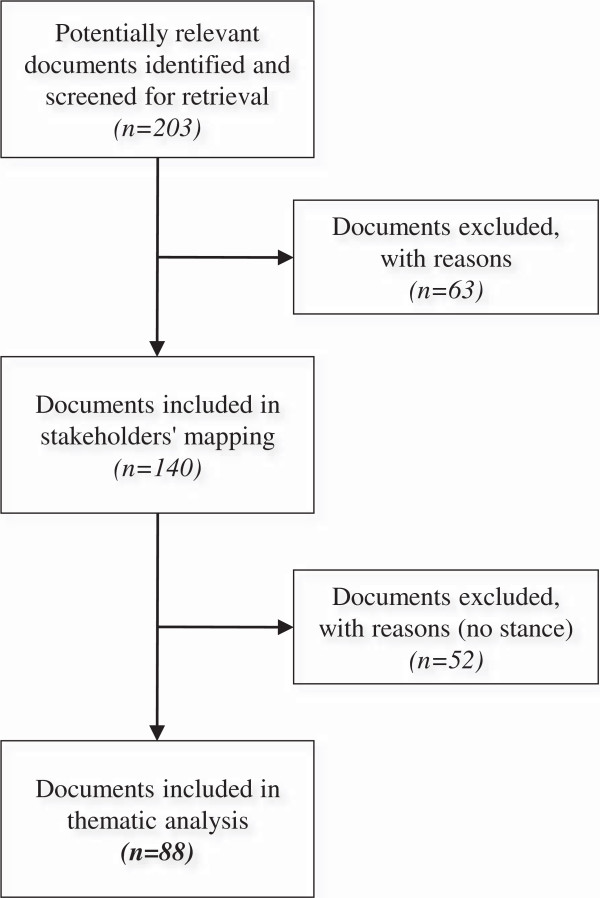
Selection process of the documents.

We identified 56 GHAs: 18 were intergovernmental organizations, 10 were government agencies, 8 were international non-governmental organizations and 20 were working groups and transnational networks. We screened 203 documents meeting the inclusion criteria 1 to 4 for all GHAs, with the exception of the African Development Bank and International Health Partnership + for which we could not find any relevant documents. Of these documents, 63 were excluded (Figure 2) because they did not meet the inclusion criteria 5 and 6. The stakeholder analysis was carried out using the 140 remaining documents. The list of these documents is available in the Additional file 1. Among them, 38 were published by intergovernmental organizations, 34 were published by government agencies, 27 by international non-governmental organizations, and 41 by working groups and transnational networks. The thematic analysis was performed on the 88 documents belonging to the GHAs who have addressed the issue of access to health care, user fees or exemption policies. Table 2 describes the different types of documents.

**Table 2:** Description of the documents included in the study

	**IGOs**	**GAs**	**INGOs**	**NW/WGs**	**TOTAL**
*Total number of selected documents*	38	34	27	41	140
*Total number included in thematic analysis*	25	10	26	27	88
*Nature of documents*					
Action plan / strategy	14	9	0	3	26
Call to action / lobby document	1	0	23	15	39
Position paper / statement of principles	5	0	2	3	10
Report	5	1	1	6	13
*Topic of documents*					
Health (general)	8	3	1	3	15
MDGs	3	0	0	7	10
User fees / access to healthcare	1	0	9	0	10
Poverty / development	2	5	2	7	16
Specific health issue	6	2	14	10	32
Other	5	0	0	0	5

**Figure 3:**
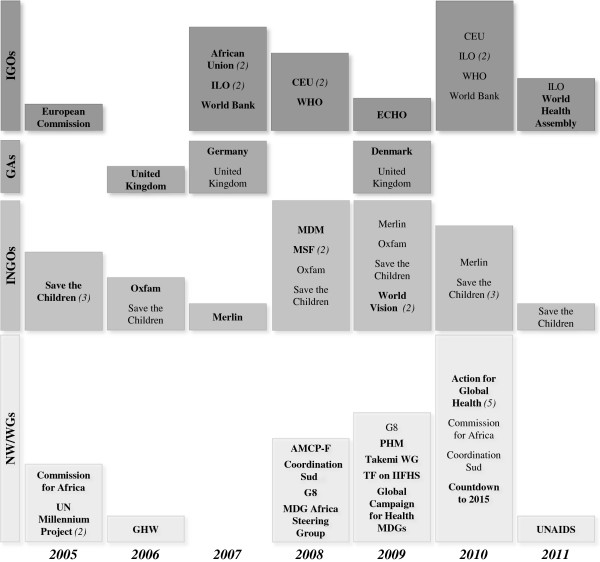
**Distribution of GHAs by year of publication of documents.** This figure shows the distribution of GHAs by year of publication of documents in which they state that they are in favor of free care at the point of delivery or in favor of abolition of user fees. The size of each year block is proportional to the number of documents published that year. When the number of documents published by a GHA is more than one, the total number is indicated in brackets. The name of the GHA is highlighted when it is the first time it takes a positive stance from 2005.

The majority of the documents in which the GHAs took a stance in favor of free care or in favor of abolishing user fees were published between 2008 and 2010 (Figure 3). Between 2005 and 2007, 19 documents (29%) were published, six of these (32%) by INGOs, while 43 (66%) were published between 2008 and 2010.

### What are GHAs’ positions on user fees?

The GHAs that took a stance in the debate are for the most part in favor of the abolition of user fees or in favor of free care at the point of delivery. Indeed, 30 of them (55%) fall into the category “*Positive stance*” (Table 3). No GHA was in favor of user fees or against free care at point of service. However, 43% of the GHAs remain silent about the issue: either they do not address the issue (30%), or they discuss it in a neutral manner (13%). A case in point is UNAIDS, a United Nations partnership devoted to the issue of HIV/AIDS, which describes user fees as a barrier to access to care, without taking a stance:

“*The costs of accessing services*, *including visit fees and transport costs*, *can also be an important barrier*, *especially among food*-*insecure people*.” (p.38) [34]

**Table 3:** Distribution of GHAs according to their position in the health user fee debate

	**No stance n = 16 (30%)**	**Neutral stance n = 7 (13%)**	**Negative stance n = 0**	**Positive stance n = 30 (55%)**	**Nuanced stance n = 1 (2%)**
**IGOs**	UNDP	Asian Development Bank		African Union	The World Bank
	UNFPA	European Parliament		Council of the European Union	
	UN General Assembly	Inter-American Development Bank		ECHO	
	UNICEF	OECD		European Commission	
	WAHO			International Labour Organization	
				World Health Assembly	
				WHO	
**GAs**	France	Sweden		Denmark	
	Canada			Germany	
	Japan			UK	
	Netherlands				
	Norway				
	USA				
**INGOs**	Gates Foundation			MDM	
	Rockefeller Foundation				
				Merlin	
				MSF	
				Oxfam	
				Save the Children	
				World Vision	
**NW/WGs**					
* GAs and IGOs*				Commission for Africa	
				G8	
				MDG Africa Steering Group	
				The Global Campaign for the Health MDGs	
				UNAIDS	
*INGOs*	InterAction			Action for Global Health	
	The Global Call to Action Against Poverty			Action mondiale contre la pauvreté	
				Coordination Sud	
				Global Health Watch	
*Other*	Global Health Council	The Global Coalition on Women and AIDS		Countdown to 2015	
		P4H		People' Health Movement	
				Taskforce on Innovative International Financing for Health Systems	
				Task Force on Global Action for Health System Strengthening	
				UN Millennium Project	

Only one GHA, the World Bank, is both in favor of user fees and in favor of free care at the point of delivery (Table 3):

“[…] *user fees have a role to play as copayment when there is evidence of excess demand*. […] *Upon client*-*country demand*, *the Bank stands ready to support countries that want to remove user fees from public facilities* […]” (p.50) [35]

There are more INGOs that take a stance in favor of free health care or against health user fees (75%) than IGOs and GAs (respectively 41% and 30%). Similarly, GAs are more likely to fall into the categories “*No stance*” or “*Neutral stance*” (70%), whereas two INGOs (25%) fall into one of these two classes.

### What characterizes the “positive stance” of GHAs?

In general, GHAs who take a stance in favor of free care or in favor of the abolition of user fees specify the populations or the type of services for which they are in favor of such measures. Documents in which GHAs take a stance often deal with mother and child health [35-37]. Some documents also deal with one or the other of these populations [34,38,39] or with poor and vulnerable populations [40-42].

With regard to the type of health services for which user fees could be abolished according to the GHAs, the following are listed: basic health care [43-46], essential health care [47-49], and primary health care [42,50,51]. GHAs also discuss HIV/AIDS prevention, treatment and care [52,53] and malaria [54]. The Global Call to Action against Poverty - France specifically mentions long-term illness [55]. Finally, some GHAs are in favor of free care for a combination of populations and services, such as basic health care for children under 5 [56,57].

Some GHAs sometimes formulate conditions in which they support free health care: the World Bank [35,58], ECHO [59], World Vision [36], Merlin [38], G8 [56,60]. For example, ECHO stipulates that free health care must be guaranteed in emergency situations [59]. The World Bank identifies three conditions:

“[…] (*a*) *the lost revenue from user fees can be replaced with resources that reach the facilities in a timely and fiscally sustainable manner over the long*-*term*; (*b*) *effective public financial management systems ensure that such financial transfer replacements will effectively reach the health facilities that need them the most and in the context of the appropriate incentive framework for the provision of services to the poor*; *and* (*c*) *that resource replacements will be used to pay for the delivery of effective quality services for the poor*, *provided at the health facilities*.” (p.50) [35]

In addition, not all GHAs formulate their stance as directly as Save the Children and some other INGOs who do not hesitate to call for the abolition of user fees: “*In order to save the lives of children and enable the poorest families to get the care they need*, *user fees must be abolished* […]” (p.3) [61]. For example, the Danish cooperation, while in favor of free care at the point of care, emphasizes the need to promote alternative financing mechanisms:

“*An important strategy towards improved coverage and universal access to health services is to reduce out*-*of*-*pocket spending and ensure social protection against catastrophic health expenditures*. *A mix of financing mechanisms is needed in most countries in order to achieve this aim*. […] *abolition of user fees is a strategy that cannot guarantee quality of service and be a stand*-*alone strategy*.” (p.15) [62]

The documents from intergovernmental organizations often mentioned the idea that user fees should be avoided, while suggesting other financing mechanisms, rather than removing them outright. This is particularly the case of the African Union [63], Council of the European Union [64], the World Health Assembly [65] and WHO [50,66].

Detailed characteristics on GHAs’ suggested measures, as well as target populations and services, are available in the Additional file 2.

### What arguments do GHAs use to justify their stance?

International non-governmental organizations are more likely to condemn user fees than other GHAs who might have previously promoted and supported their implementation. The discourse of intergovernmental organizations more often calls on a set of universal values such as fairness, justice, or the right to health (see below). Their discourse is not as committed as that of INGOs and they rarely highlight the disadvantages of user fees, such as the African Union:

“*In exploring additional sources of revenue countries should work towards a solidarity model within a framework of equity*, *seeking to implement pre*-*payment systems to avoid user fees at the time that care needs to be sought*.” p.11 [67]

Notable exceptions among intergovernmental organizations are WHO and ECHO, who are true campaigners against user fees.

Regarding the nature of the arguments, we identified three main types of arguments (Table 4): economic arguments, moral and ethical arguments, and pragmatic arguments. Economic arguments are built around two rationales. The first one concerns the catastrophic health expenditures that households incur specifically as a result of user fees. These catastrophic expenditures would lead to the further impoverishment of the poorest households: “*Where user fees exist*, *those who struggle to pay can be pushed further into poverty or debt*” (p.1) [61]. Some GHAs also present their views in the context of the fight against poverty and the promotion of growth, as indicated by the titles of documents, including one from the Council of the European Union: “*The EU as a global partner for pro*-*poor and pro*-*growth development*” [52]. The second rationale relates directly to the effectiveness of the health system. Indeed, user fees would not be “*the best return on investment*” (p.25) [68] and their abolition would improve the cost-effectiveness of health services by promoting the use of services (p.69) [69].

**Table 4:** Distribution of GHAs according to the nature of their arguments

**Type of arguments**	**IGOs**	**GAs**	**INGOs**	**NWs/WGs**	**TOTAL**
1) Economics	6	2	6	6	**20**
2) Moral and ethics					
* Equity*	6	2	4	2	**14**
* Ethics*	5	1	3	4	**13**
* Human rights*	2	1	4	3	**10**
3) Pragmatic					
* Health preoccupations*	7	2	6	6	**21**
* MDGs*	4	2	4	11	**21**

With regard to moral and ethical arguments, equity is the reason most frequently cited by GHAs to justify their stance in the debate on user fees, and this view is relatively homogeneous. On one hand, user fees appear to be the most inequitable financing mechanism of health systems: “*Direct payments are the least equitable form of health funding*.” (p.42) [66]. On the other hand, they are considered to have failed to ensure equitable access to health [44].

User fees are also condemned because they accentuate gender inequalities as women often need to ask for money from their husbands before seeking care [38,68]. Other ethical arguments are used by GHAs. They declare that user fees are a source of exclusion and discrimination [57,70,71], that they are unfair [61,63] or “*intolerable*” [59], and that they pose a threat to life itself [72,73].

Some GHAs, especially international non-governmental organizations, take a principled stand in favor of the right to health which frames their point of view on free care: “*Commitments to child survival are legal obligations enshrined in a series of international human rights instruments*” (p.1) [74]. Lastly, one NW/WG – the People’s Health Movement – considers that “*health services must be democratic*” (p.11) and should therefore be free of charge [75].

In the case of pragmatic arguments, health concerns of populations are the most frequently cited. Indeed, for GHAs, user fees are a barrier that prevents people, especially the poorest, from accessing health care [66,73]. This barrier should be removed in order to improve access to health care and use of health services [37,45,65], as well as early diagnostics [51], to eliminate certain diseases [45,54,76], to improve quality and coverage of health services [38,51], and ultimately to save lives [36,43,77]. The second argument used by GHAs concerns the health Millennium Development Goals. According to the vast majority of GHAs, removing user fees would contribute to achieve the health Millennium Development Goals:

“*In the absence of more pro*-*poor funding policies being put in place in these countries* – *including providing care free at the point of use* – *the current lack of progress towards MDG 4 looks set to continue*.” (p.36) [78]

These arguments appeal to several concepts and ideas like poverty, development, equity, or the right to health, for which GHAs do not specify any definition in the documents included in the study.

To conclude, we note that several GHAs strengthen their argument by using scientific literature (n = 14), gray literature (n = 20), or their own research results or experience (n = 7).

## Discussion

### Key messages

Our study shows that promoting user fees as a way of financing health systems of LMICs is no longer the trend. There is indeed a consensus among GHAs who take a stand, at least from a rhetorical point of view, in favor of avoiding user fees at the point of delivery. User fees are deemed not only as the most inequitable means of health system financing, but also as a major barrier to access to care. However, the consensus is less obvious on the action. Most GHAs suggest abolishing user fees or establishing free access to health services. Several others, including intergovernmental organizations, avoid mentioning free healthcare; rather, they emphasize the need to find alternative mechanisms of financial protection, such as social protection [64,79] or pre-payment mechanisms [63,65]. As shown in the Additional file 2, beneficiaries of these measures are often identified as the most vulnerable and the poorest and coincide with the target beneficiaries of the health Millennium Development Goals (mothers, children under 5, people living with HIV/AIDS). This is consistent with the statements made by some GHAs that user fees hinder the achievement of the Millennium Development Goals by slowing access to health care.

Our study also shows that international non-governmental organizations are more likely to take a stand than other GHAs. Most [80-83] have an advocacy mission, which leads them to take sides on diverse development or humanitarian issues, such as access to care. They are also direct witnesses of the problem of access to care for vulnerable populations. In contrast, few government agencies take a stand, probably because of their position in the global agora. Indeed, government agencies are responsible for implementing the foreign policy of their country, whose first aim is to defend its national interests on the international scene [84,85]. Each undertaking is part of a complex diplomatic logic [85], especially as some are among the largest donors in the field of health.

### History of changes

While some international non-governmental organizations have been defending the principle of free care for a long time, this is not the case of government agencies and intergovernmental organizations that have sometimes contributed - directly or indirectly - to the implementation of user fees. Indeed, UNICEF and WHO influenced the Bamako Initiative, which promoted user fees in LMICs. At that time, the World Bank was becoming influential in the health sector, transforming power relations on the international health scene [86-88]. Although it was not involved in the development of the Bamako Initiative, it pursued structural adjustment programs in developing countries since the early 1980s that aimed to reduce public spending in the social sectors. These programs contributed to the expansion of the Bamako Initiative. The diversification and expansion of the World Bank to the health sector influenced the nature of health financing reforms, leading to the “*fabrication of a consensus*”, as put by Serre and Pierru [88].

This movement was part of a broader discussion about health system financing in Western countries and in LMICs, which started in the early 1990s. In this way, “[…] *the propagation of HCF* (*health care financing*) *has been* […] *shaped by the building of a consensus across different institutions and national settings*, *defining the* '*problem*' *of HCF and potential solutions*” [87]. Through its financial strength and technical expertise, the World Bank became the leader on the global health scene [89], defining new norms and rules for international cooperation organizations, academic institutions and policy makers of LMICs who were dependent on international aid [86]. A paradigm was born and contributed to influence the vast majority of health care financing reforms in LMICs, particularly on the African continent.

The discourse on health care financing is characterized by a change in the stance of major GHAs, including intergovernmental organizations. Indeed, between the stance of the World Bank in 1987 and its stance twenty years later [35], the dominant principle of user-pays has fizzled out. The recent speech by its president Jim Yong Kim at the World Health Assembly [90] in May 2013 gives effect to this change. He claimed there: “*Anyone who has provided health care to poor people knows that even tiny out*-*of*-*pocket charges can drastically reduce their use of needed services*. *This is both unjust and unnecessary*.”

### Incremental reform or fundamental change?

Is this change *incremental* or *fundamental*? As opposed to incremental change, fundamental or paradigmatic change is “*a fundamentally new direction* […], *also understood as signifying the emergence of a new paradigm or way of thinking about a policy issue*.” [91]. If our study shows the existence of a consensus, it also shows that some intergovernmental organizations and working groups and transnational networks have a nuanced stance and abstain, in particular, from promoting the abolition of user fees. They have a moderate discourse in which one perceives a tension between the desire to support the abolition of direct payments, for the sake of fairness and social justice, and the need to provide solutions to ensure the sustainability of health care financing. The same tension exists in the discourse of health staff, particularly in Mali, who admit that user fees are a barrier to access to health care for the poor, while remaining cautious about possible alternatives [92]. Therefore, the consensus may be more about the need to find alternative ways to finance equitable health systems, such as universal health coverage.

In addition, we may legitimately ask whether certain intergovernmental organizations’ statements are not empty statements of principle. Indeed, speech façades, that use the vocabulary promoted by civil society in particular, are no stranger to international health policy [93]. For example, the goal of fairness that is shared by all GHAs and that helps justify their stance is never defined. The more systematic presence of this objective in the discourse of GHAs is certainly a step towards the establishment of common values within the international community [94]. Yet, the risk that it becomes a slogan for some GHAs is present.

### Evidence and networks: the drivers of change

The change of GHAs’ stance can be explained by the coexistence of several factors. We believe that the production and dissemination of scientific evidence on user fees and the influence of some networks of actors in global health have contributed to this change.

Since the 1990s, the evidence-based approach has gained interest, particularly in the area of political decision-making [95]. This approach aims to encourage policy-makers to take scientific evidence into consideration when developing new policies. International organizations have undertaken to contribute to the production of knowledge, either through their own publications [88,95], or through the financing of research in the academic world [87]. This evidence could allow some intergovernmental organizations to justify particular discourses and promote specific policies, from a strategic and argumentative perspective [88].

Given the considerable challenge of health care financing reforms, and in order to inform policy makers and the public about the effects of these reforms, many researchers have focused on the impact of user fees on utilization of health services by populations [15]. For their part, INGOs, especially Save the Children, have capitalized on their field experience to produce information on the effects of user fees on access to care for vulnerable populations [48,96]. Despite the methodological limitations of these studies, the results mostly demonstrated a negative impact of user fees on access to health care for the poor [11]. This abundance of scientific evidence and field experience may have contributed to change the position of GHAs. It also strengthened the committed discourse of INGOs, as shown by the use of scientific literature in their documents. If evidence could not be the single factor changing the position of intergovernmental organizations, it certainly contributed to the already strong debate between supporters and critics of user fees, while casting doubt on these cost recovery policies in the minds of LMICs policy makers.

Knowledge can contribute to political change, but only as the instrument of change that actors must use. These actors, whether they are individual or collective, have beliefs and values. They have goals they seek to achieve through the mobilization of resources and they are constrained by rules and institutions [97]. Advocacy networks offer an interesting perspective for understanding the involvement of different actors in policy changes over long periods [98]. These networks consist of “*persons holding a variety of positions* (*elected officials*, *interest group leaders*, *researchers*) *who share a particular belief system* – *i*.*e*., *a set of basic values*, *causal reasoning*, *and perceptions of problems* – *and who show a significant degree of coordinated activity over time*”. In this perspective, ideas have an important role in the process of change and elaboration of public policy at the national level. Two types of networks seem to have played a role in the political change of health care financing in LMICs: global policy networks, particularly transnational political elite [87], and transnational advocacy networks [99].

Lee et Goodman [87] trace the path of health care financing reforms and show the role of global policy networks in building consensus: “*This consensus has been achieved through a range of research and training initiatives*, *project funding*, *the career movement of individuals*, *and other forms of collaborative work across higher*- *and lower*-*income countries*, *health economics and public health*, *and public and private sectors*." The authors highlight the "*transatlantic divide*” between American schools of thought, which are in favor of user fees, and European schools, which are rather unfavorable to user fees. The scope of this study ends in the 2000s and offers a historical perspective that is necessary to understand how the terms of the debate on user fees in LMICs evolved. For further analysis, we could focus on elements recognized as having fostered the emergence of a consensus, including how European schools of thought have gained legitimacy, to the point of participating in reversing dominant positions.

Transnational advocacy networks [99] differ from advocacy networks not only by their transnational character, but also by their activism. A transnational advocacy network “*includes those relevant actors working internationally on an issue*, *who are bound together by shared values*, *a common discourse*, *and dense exchanges of information and services*.” Among these networks, INGOs play a central role, as they are able to initiate actions and put pressure on key international stakeholders. Indeed, INGOs “*participate in the agenda setting of international issues*, *in the decision*-*making process and in the implementation of international action programs*” [100]. Several INGOs have published a large number of reports showing the negative effects of user fees on access to care for the poor, while putting pressure on their government and / or intergovernmental organizations to make them take a clear position in favor of free care at the point of delivery. Transnational advocacy networks have several levers to influence policy decision-making [99]: “*information politics*” which is the capacity to quickly mobilize politically relevant information; “*symbolic politics*” which is the ability to use symbols or striking stories to give meaning to action; “*leverage politics*” which is the ability to solicit more powerful stakeholders to influence a situation; “*accountability politics*” which is the ability to compel actors to comply with their declaration of intent. We think that the study of these tactics would allow a better understanding of the influence that these actors have had in changing the stance of intergovernmental organizations and government agencies with respect to user fees in LMICs.

Beyond these two drivers of change, our study suggests a third driver: the LMICs themselves. We note that the publication of documents in which GHAs take a stand in favor of free care or the abolition of user fees increases between 2008 and 2010. It is during this period that influential stakeholders such as the WHO, the European Commission, the G8 and several working groups and transnational networks take a stand in the debate. However, these documents follow the decision of several LMICs to implement user fee exemption policies. Indeed, Ghana and Burkina Faso opted for exemption policies for maternal health in 2003, followed in 2005 by Niger, Mali and Senegal [19]. This suggests that most GHAs may have felt they had to take a stand with regard to the policies initiated by LMICs. However, the multiplicity of exemption measures since 2009, particularly in West Africa, suggests that the stance of GHAs has in turn prompted other LMICs such as Côte d'Ivoire, Togo and Sierra Leone to implement exemption policies.

### From words to deeds

Whatever the nature of the change that is taking place on the international health scene, the stance of GHAs alone is ineffective in improving the health of populations. Even if “*change may be symbolic before being concrete due to the performative dimension of political discourse*” [100], it must be transformed into action to last. The first to take action were international non-governmental organizations, including MSF and MDM. Their documents show their experiences in implementing free health care at the local level. These experiences provided them with arguments for their lobby campaigns. More recently, intergovernmental organizations have pressured governments of LMICs to introduce user fee exemption measures. As an example, in Burkina Faso, the World Bank has supported the implementation of the subsidy for emergency obstetric and neonatal care [101]. In Niger, the Bank has subordinated loans to the introduction of free health care for children under 5 [102]. However, the Bank’s conditions were not followed by technical or financial support. In Niger, it has not provided the necessary financial support to ensure the smooth functioning of the exemption measures it advocated. This has forced the country to turn to other donors, such as the French cooperation [102] which allowed a part of its aid to be used to fund free care for children under 5. The limits of their actions could explain why intergovernmental organizations tend not to mention free care or exemption policies as possible solutions, and merely highlight the inequitable nature of user fees.

These examples illustrate the difficulties faced by LMICs to implementing user fee exemption policies, without the support of donors. Indeed, although many exemption policies were implemented in several sub-Saharan African countries, their outcomes in terms of service utilization were marred by problems of implementation. A lack of preparation, both operational and financial, has been highlighted in many studies [18,103]. A major repercussion was the return of illegal user fees [104-106], which may counteract the expected policy effects. Furthermore, exemption policies’ sustainability is far from assured [107], and out-of-pocket payments remain an issue for those who do not benefit from these policies or have to pay for transportation, medicine, etc. [66]. Thus, although they have generally shown their potential to improve health services utilization [108-110], user fee exemption policies are rarely promoted as a way to ensure equitable access to healthcare. However they are increasingly seen as a step towards universal health coverage [20,107,111]. In this context, the debate now seems to be more about the most effective and equitable mechanisms to achieve universal health coverage in LMICs.

### Limits

Although we conducted the study so as to limit bias, including interpretation bias, our work has some limitations that are inherent to the research design. First, because we used only secondary data, we limited our analysis to the official and public discourse of major stakeholders. However, as shown in the case of Niger, where France supported the user fee exemption policy, the absence of documents addressing the issue of user fees by some GHAs does not mean that there are no positions taken on “the ground”. Similarly, the financial or technical support by a GHA for a national user fee exemption policy or a local free care program does not necessarily mean that it takes a stand against health user fees or for universal free care. Such actions may be motivated by contextual considerations that are political, social or health-related.

In addition, it is possible that certain documents were not found, a major challenge being to ensure the completeness of the data collection strategy. We recall in this respect that the documents marked “*the views expressed in this paper are solely those of the author*” and similar were excluded. It is also possible that other GHAs not included in our initial sample expressed opinions on the issue of user fees. However, we could not trace their presence in the debate. Indeed, the multiplicity of stakeholders makes it difficult to ensure their full representation. Moreover, the data collection strategy ended in mid-2011; other documents may have been published since and state, specify or modify the stance of some GHAs. Ensuring that findings are up-to-date can be challenging when dealing with global policy issues that are very contemporary and are under close scrutiny.

Finally, the objective of this study was to identify and analyze the public position statements of GHAs in the debate on health user fees. It would have been particularly interesting to understand the evolution of the change in the stances of GHAs in this debate and analyze how it relates to the international debate on healthcare financing. However this is beyond the scope of our study.

## Conclusion

Our study is one of the few to analyze the stance of GHAs on a global health policy issue based on the analysis of public documents. It shows that there is a consensus on the need to make health care free at the point of delivery in LMICs to ensure equitable access for all. The consensus seems to follow, not precede, the establishment of user fee exemption policies in some LMICs. While universal health coverage becomes the new objective in global health policy, free care at the point of delivery is far from being a reality for many citizens in LMICs. This is why GHAs, and especially intergovernmental organizations, should not settle for a speech front and must translate their words into action. Intergovernmental organizations and government agencies who have the necessary means must ensure both technical and financial support to the governments of LMICs committed to implement user fee exemption policies. As for the scientific community, we should continue our work to improve GHAs’ understanding of challenges, successes and obstacles faced by governments when implementing such reforms. We also need to better understand the historical evolution of the debate on user fees, as a way to understand global governance and how it influences public health policies in LMICs.

## Abbreviations

AFD: Agence Française du Développement (French Development Agency); AMCP-F: Action Mondiale Contre la Pauvreté – France; CEU: Council of the European Union; CIDA: Canadian International Development Agency; DANIDA: Danish International Development Agency; DFID: Department for International Development – United Kingdom; ECHO: Humanitarian aid Department of the European Commission; GA: Government Agency; GHA: Global Health Actor; GHW: Global Health Watch; GIZ: Germany Development Cooperation; INGO: International Non-government Organization; IGO: International Government Organization; ILO: International Labour Organization; JICA: Japan International Cooperation Agency; LMIC: Low- and middle-income country; MDG: Millennium Development Goal; MDM: Médecins du Monde; MSF: Médecins Sans Frontières; NW/WGs: Network / Working group; OECD: Organization for Economic co-operation and Development; P4H: Providing for Health; PHM: People’s Health Movement; TF on IIFHS: Taskforce on innovative international financing for health systems; UNAIDS: Joint United Nations Programme on HIV/AIDS; UNDP: United Nations Development programme; UNFPA: United nations Population Fund; UNICEF: United Nations Children's Fund; USAID: United States Agency for International Development; WAHO: West African Health Organization; WHO: World Health Organization.

## Competing interests

The authors declare that they do not have any competing interests.

## Authors’ contributions

Based on the original idea of VR and under VR’s supervision, ER conducted the study. ER and VR searched for potentially relevant documents and designed the decision tree. ER carried out the analysis and drafted the first version of the manuscript. VR critically revised the successive versions of the manuscript. All authors read and approved the final manuscript.

## Authors’ information

ER is a Ph.D. candidate in public health at Montreal University (Canada). She is a senior fellow of the Global Health Research Capacity Strengthening Program (GHR-CAP). ER graduated from the Institute of Political Studies of Toulouse (France) and from the Institute for the Study of Economic and Social Development from Université Paris 1 (France).

VR is an associate professor at Montreal University (Canada) in the Department of social and preventive medicine. He is a researcher at the Research Center of Montreal University Hospital Center (CRCHUM).

## Supplementary Material

Additional file 1 List of all included documents. Click here for file

Additional file 2 Characteristics of the positive or nuanced stance of GHAs. Click here for file
