# Supplementary material for: Global health actors no longer in favor of user fees: a documentary study
Source: Global Health. 2013 Jul 26;9:29. doi: 10.1186/1744-8603-9-29 (PMC3750575; doi:10.1186/1744-8603-9-29)
Supplement: Additional file 1 — List of all included documents. [file 1744-8603-9-29-S1.pdf]

DOCUMENTS PUBLISHED AND CREDITED BY GLOBAL HEALTH ACTORS (2005-2011)

Note: URL links were updated on October 9, 2012 or later. Some original URL links are missing. Documents can be obtained upon request to the lead author (emilie.robert.3@umontreal.ca).

1. Action for Global Health. (2009). *Health in crisis: Why, in time of economic crisis, Europe must do more than ever to achieve the health Millennium Development Goals*. Brussels. Retrieved from **http://www.actionforglobalhealth.eu/uploads/media/AFGH__Health_in_Crisis__Report2009_.pdf**
2. Action for Global Health. (2009). *Right to health and the health MDGs*. Brussels. Retrieved from **http://www.actionforglobalhealth.eu/fileadmin/user_upload/_temp_/Right_to_Health_position_paper_AFGH_01.pdf**
3. *Action for Global Health. (2010). *2010 reality check: Time is running out to meet the health MDGs*. Brussels. Retrieved from **http://www.actionforglobalhealth.eu/fileadmin/user_upload/_temp_/Action_Aid__WEBLORES_.pdf**
4. *Action for Global Health. (2010). *Achieving the health MDGs by 2015: What the EU needs to do!* Brussels. Retrieved from **http://www.actionforglobalhealth.eu/fileadmin/user_upload/_temp_/AFGH_EU-Roadmap_NEW.pdf**
5. *Action for Global Health. (2010). *Action for Global Health recommendations for the Council Conclusions on the European Commission' s Communication on the EU Role in Global Health*. Brussels. Retrieved from **http://www.globalhealthguide.eu/files/AFGH_Open_Letter_Global_Health_Communication.pdf**
6. *Action for Global Health. (2010). *Improving the health of children across the world needs to be made a priority by governments*. Strasbourg. Retrieved from **http://www.globalhealthguide.eu/files/MDG4_CSOstatement_FINAL.pdf**
7. *Action for Global Health, & Hart, M. (2010). *Dear member of the Development Cooperation Working Group*. Brussels. Retrieved from **http://www.globalhealthguide.eu/files/AfGH_recommendations_for_the_MDG_summit.pdf**
8. *Action mondiale contre la pauvreté. (2008). *Health for development: challenges and responsibilities. At the midway point in the Millennium development goals, where do we stand on health? The French civil society recommendations*. Paris. Retrieved from **http://dev.actionmondialecontrelapauvrete.org/wp-content/uploads/2010/05/AMCP2007_Position_Santé_FR.pdf**

9. *African Union. (2007). *The new partnership for Africa's development (NEPAD): health strategy*. Addis Ababa. Retrieved from **http://www.iag-agi.org/bdf/docs/framework_-_nepad_health_strategy.pdf**

10. *African Union. (2007). *Africa Health Strategy: 2007 - 2015*. Addis Ababa. Retrieved from **http://www.africa-union.org/root/UA/Conferences/2007/avril/SA/9-13 avr/doc/en/SA/AFRICA_HEALTH_STRATEGY.pdf**

11. African Union. (2010). *Actions on maternal, newborn and child health and development in Africa by 2015*. Kampala. Retrieved from **http://www.unfpa.org/webdav/site/global/shared/documents/news/2010/kampala_au_assembly_dec.pdf**

12. Agence française de développement. (2011). *Projet d'orientation stratégique 2007-2011 de l'Agence française de développement*. Paris. Retrieved from **http://www.afd.fr/webdav/site/afd/shared/ELEMENTS_COMMUNS/pdf/POS 2 2007-2011.pdf**

13. *Asian Development Bank. (2005). *Development, poverty and HIV/AIDS: ADB's strategic response to a growing epidemic*. Metro Manila. Retrieved from **http://www.adb.org/sites/default/files/pub/2005/in90-05.pdf**

14. *Asian Development Bank. (2008). *An operational plan for improving health access and outcomes under Strategy 2020*. Metro Manila. Retrieved from **http://www.adb.org/sites/default/files/pub/2008/Operational-Plan-For-Health.pdf**

15. Asian Development Bank. (2008). *Strategy 2020: The long-term strategic framework of the Asian Development Bank 2008-2020*. Metro Manila. Retrieved from **http://www.adb.org/sites/default/files/Strategy2020-print.pdf**

16. *Asian Development Bank. (2011). *Strategic Directions Paper on HIV / AIDS 2011 – 2015*. Metro Manila. Retrieved from **http://www.adb.org/sites/default/files/strategic-directions-hiv-aids-2011-2015.pdf**

17. Bill and Melinda Gates foundation. (2010). *Global health program*. Washington, D.C. Retrieved from **http://www.gatesfoundation.org/global-health/Documents/global-health-program-overview.pdf**

18. Canadian International Development Agency. (2006). *Sustainable Development Strategy 2007-2009*. Ottawa. Retrieved from **http://www.acdi-cida.gc.ca/INET/IMAGES.NSF/vLUImages/Sustainable_development/$file/Sustainable%20Development%20Strategy2007–2009.pdf**

19. Canadian International Development Agency. (2009). *Securing the future of children and youth: CIDA's children and youth strategy*. Ottawa. Retrieved from **http://www.acdi-cida.gc.ca/INET/IMAGES.NSF/vLUImages/Youth-and-Children/$file/children-youth-strategy-e.pdf**

20. *Commission for Africa. (2005). *Our common interest: report of the Commission for Africa*. London. Retrieved from **http://ocpa.irmo.hr/resources/docs/Commission_for_Africa_Report-en.pdf**

21. *Commission for Africa. (2010). *Still our common interest: commission for Africa report 2010*. London. Retrieved from **http://www.commissionforafrica.info/wp-content/uploads/2010/09/cfa-report-2010-full-version.pdf**

22. *Commission of the European Communities (CE). (2005). *A European Programme for Action to Confront HIV/AIDS, Malaria and Tuberculosis through External Action (2007-2011)*. Brussel. Retrieved from **http://eur-lex.europa.eu/LexUriServ/LexUriServ.do?uri=COM:2005:0179:FIN:EN:PDF**

23. Commission of the European Communities (CE). (2006). *Investing in people: Communication on the thematic programme for human and social development and the financial perspectives for 2007-2013*. Brussels. Retrieved from **http://eur-lex.europa.eu/LexUriServ/LexUriServ.do?uri=COM:2006:0018:FIN:EN:PDF**

24. Coordination Sud. (2007). *Préparation du G8 de Heiligendamm 6/8 Juin 2007 : Propositions des ONG françaises*. Paris. Retrieved from **http://www.france.attac.org/archives/spip.php/spip.php?article7041&artpage=0-0**

25. *Coordination Sud. (2008). *Préparation du G8 de Hokkaido, Toyako, Japon, 7-9 Juillet 2008 : Propositions des ONG françaises.* Paris. Retrieved from **http://www.ong-ngo.org/old/IMG/pdf/Positions_Coordination_SUD_G8_2008.pdf**

26. *Coordination Sud. (2010). *Proposition des ONG françaises. G8 Muskoka - 25 et 26 juin 2010. G20 Toronto - 26 et 27 juin 2010*. Paris. Retrieved from **http://www.coordinationsud.org/wp-content/uploads/document_position_g8_g20.pdf**

27. Coordination Sud & Ferrier, F. (2004). *Positions des ONG française, G8 2003, Evian, France*. Paris. Retrieved from **http://doc-aea.aide-et-action.org/data/admin/position_des_ong_francaises_au_g8_d_evian.pdf**

28. *Council of the European Union. (2008). *Council Conclusions on the promotion and protection of the rights of the child in the European Union's external action - the development and humanitarian dimensions*. Brussels. Retrieved from **http://www.eu2008.si/en/News_and_Documents/Council_Conclusions/May/0526_GAERC-pravice_otrok.pdf**

29. *Council of the European Union. (2008). *The EU as a global partner for pro-poor and pro-growth development: EU Agenda for Action on MDGs*. Brussels. Retrieved from **http://register.consilium.europa.eu/pdf/en/08/st11/st11096.en08.pdf**

30. *Council of the European Union. (2010). *Council conclusions on the EU role in Global Health.* Brussels. Retrieved from **http://www.consilium.europa.eu/uedocs/cms_data/docs/pressdata/en/foraff/114352.pdf**

31. *Countdown to 2015. (2010). *Countdown to 2015 decade report (2000-2010) with country profiles: Taking stock of maternal, newborn and child survival*. Geneva. Retrieved from **http://whqlibdoc.who.int/publications/2010/9789241599573_eng.pdf**

32. DANIDA. (2007). *A world for all: Priorities of the Danish Government for Danish Development Assistance 2008-2012*. Copenhagen. Retrieved from **http://amg.um.dk/en/~/media/amg/Documents/Overall%20Policies/Priorities%20of%20the%20Danish%20Government/2007%20A%20World%20for%20All/AWorldForAll2007.ashx**

33. DANIDA. (2010). *Freedom from poverty, freedom to change: Strategy for Denmark's development cooperation*. Copenhagen. Retrieved from **http://um.dk/en/~/media/UM/English-site/Documents/Danida/Goals/Strategy/Freedom from poverty.ashx**

34. *Department for International Development. (2005). *Social Transfers and Chronic Poverty: emerging evidence and the challenge ahead*. London. Retrieved from **http://www.unicef.org/socialpolicy/files/social-transfers_and_chronic_poverty.pdf**

35. *Department for International Development. (2006). *Eliminating world poverty: making governance work for the poor. A White Paper on International Development* (DFID.). London: The Stationery Office. Retrieved from **http://www.official-documents.gov.uk/document/cm68/6876/6876.pdf**

36. *Department for International Development. (2007). *Working together for better health*. London. Retrieved from **http://www.who.int/pmnch/activities/advocacy/workingtogether.pdf**

37. *Department for International Development. (2009). *Eliminating World Poverty: Building Our Common Future*. London: TSO (The Stationery Office). Retrieved from **www.infodev.org/en/Document.671.pdf**

38. *ECHO. (2009). *DG ECHO position paper on user fees for primary health services in humanitarian crises*. Brussels. Retrieved from **http://ec.europa.eu/echo/files/policies/sectoral/health_2009_note_on_user_fees.pdf**

39. European Commission. (2007). *Investing in people: Strategy Paper for the Thematic Programme 2007–2013*. Brussels. Retrieved from **http://ec.europa.eu/development/icenter/repository/how_we_do_strategy_paper_en.pdf**

40. European Commission. (2010). *A twelve-point EU action plan in support of the Millennium Development Goals*. Brussels. Retrieved from **http://ec.europa.eu/development/icenter/repository/COMM_COM_2010_0159_MDG_EN.PDF**

41. *European Parliament Committee on Development. (2010). *Draft report on health care systems in sub-Saharan Africa and global health*. Strasbourg. Retrieved from **http://www.europarl.europa.eu/meetdocs/2009_2014/documents/deve/pr/819/819607/819607en.pdf**

42. *European Parliament Council Commission. (2011). *The European Consensus on Development*. Brussels. Retrieved from **http://ec.europa.eu/development/icenter/repository/european_consensus_2005_en.pdf**

43. *Federal Ministry for Economic Cooperation and Development. (2007). *Promoting Health – Fighting HIV / AIDS*. Berlin. Retrieved from **www.stoyke.com/aids/dokumente/01/018_en_bmz_gesundheit_foerdern.pdf**

44. Federal Ministry for Economic Cooperation and Development. (2008). *Development Policy Action Plan on Human Rights 2008 – 2010*. Bonn. Retrieved from **http://www.bmz.de/en/publications/type_of_publication/strategies/konzept167.pdf**

45. Federal Ministry for Economic Cooperation and Development. (2008). *Sexual and Reproductive Health and Rights, and Population Dynamics: A BMZ Policy Paper*. Bonn. Retrieved from **http://www.bmz.de/en/publications/type_of_publication/strategies/spezial149pdf.pdf**

46. Federal Ministry for Economic Cooperation and Development. (2008). *Towards One World: Development Policy White Paper*. Bonn. Retrieved from **http://reliefweb.int/sites/reliefweb.int/files/resources/16AEE43BE4026B89492575BB001A0735-Weissbuch_en.pdf**

47. Federal Ministry for Economic Cooperation and Development. (2009). *Health and Human Rights*. Bonn. Retrieved from **http://www.bmz.de/en/publications/type_of_publication/strategies/spezial165.pdf**

48. *Federal Ministry for Economic Cooperation and Development. (2009). *Sector Strategy: German Development Policy in the Health sector* (pp. 1–22). Bonn. Retrieved from **http://www.bmz.de/en/publications/type_of_publication/strategies/konzept187.pdf**

49. Federal Ministry for Economic Cooperation and Development. (2009). *Sector Strategy on Social Protection*. Bonn. Retrieved from **http://www.bmz.de/en/publications/type_of_publication/strategies/konzept190.pdf**

50. *G8. (2005). *Chair's summary, Gleneagles summit, 8 July*. Gleneagles. Retrieved from **http://www.canadainternational.gc.ca/g8/assets/pdfs/g8_Chair_Summary-en.pdf**

51. G8. (2006). *Chair's summary*. St. Petersburg. Retrieved from **http://en.g8russia.ru/docs/25.html**

52. G8. (2007). *Chair's summary*. Heiligendamm. Retrieved from **http://www.g-8.de/Content/EN/Artikel/__g8-summit/anlagen/chairs-summary,templateId=raw,property=publicationFile.pdf/chairs-summary.pdf**

53. *G8. (2008). *G8 Hokkaido Toyako Summit Leaders Declaration*. Toyako. Retrieved from **http://www.canadainternational.gc.ca/g8/assets/pdfs/G8_Hokkaido_Toyako_Summit_Leaders_Declaration-eng.pdf**

54. *G8. (2009). *Responsible leadership for a sustainable future*. L'Aquila. Retrieved from **http://www.g8italia2009.it/static/G8_Allegato/G8_Declaration_08_07_09_final,0.pdf**

55. G8. (2010). *G8 Muskoka Declaration: recovery and new beginnings*. Muskoka. Retrieved from **http://canadainternational.gc.ca/g8/assets/pdfs/2010-declaration_eng.pdf**

56. G8. (2011). *Renewed commitment for freedom and democracy*. Deauville. Retrieved from **http://www.g8.utoronto.ca/summit/2011deauville/2011-declaration-en.html**

57. Global Health Council. (2010). *Maternal, Newborn, Child and Reproductive Health: A Global Health Council Position Paper*. Washington, D.C. Retrieved from **http://www.healthynewbornnetwork.org/sites/default/files/resources/GHC_2010_position_mnchfp.pdf**

58. Global Health Council. (2011). *Malaria: A Global Health Council Position Paper*. Washington, D.C. Retrieved from **ghi.yale.edu/node/346/attachment**

59. Global Health Council. (2011). *Neglected Tropical Diseases: A Global Health Council Position Paper*. Washington, D.C. Retrieved from **ghi.yale.edu/node/359/attachment**

60. *Global Health Watch. (2006). *Global Health Watch 2005-2006: An alternative world health report*. London. Retrieved from **http://www.ghwatch.org/sites/www.ghwatch.org/files/ghw.pdf**

61. Government Offices of Sweden. (2007). *Sweden's Strategy for Multilateral Development Cooperation*. Stockholm. Retrieved from **http://www.government.se/sb/d/3365/a/97680**

62. *Government Offices of Sweden. (2008). *Global Challenges - Our Responsibility: Communication on Sweden's policy for global development*. Stockholm. Retrieved from **http://www.government.se/sb/d/9807/a/113283**

63. *Government Offices of Sweden. (2008). *The Right to a Future: Policy for Sweden's international HIV and AIDS efforts*. Stockholm. Retrieved from **http://www.regeringen.se/sb/d/5348/a/133242**

64. Government Offices of Sweden. (2011). *Saving lives and alleviating suffering: Policy for Sweden's humanitarian assistance 2010-2016*. Stockholm. Retrieved from **http://www.government.se/sb/d/3365/a/172910**

65. *Inter-American Development Bank. (2011). *Strategy on social policy for equity and productivity: Social sector*. Washigton, D.C. Retrieved from **http://idbdocs.iadb.org/wsdocs/getdocument.aspx?docnum=35802223**

66. InterAction. (2010). *Achieving the Millennium Development Goals (MDGs): The Road Forward for the U.S.* Washington, D.C. Retrieved from **http://www.interaction.org/sites/default/files/MDG_Report_0710.pdf**

67. *International Labour Office. (2010). *Achieving MDG 5 through Decent Work*. Geneva. Retrieved from **http://www.ilo.org/travail/info/fs/WCMS_141549/lang--en/index.htm**

68. *International Labour Office. (2010). *Extending social security to all: A guide through challenges and options*. Geneva. Retrieved from **http://www.ilo.org/global/publications/ilo-bookstore/order-online/books/WCMS_146616/lang--en/index.htm**

69. *International Labour Organization. (2007). *Safe maternity and the world of work*. Geneva. Retrieved from **http://www.ilo.org/wcmsp5/groups/public/---dgreports/---dcomm/---publ/documents/publication/wcms_104862.pdf**

70. *International Labour Organization. (2007). *Social health protection: An ILO strategy towards universal access to health care (a consultation)*. Geneva. Retrieved from **http://www.ilo.org/gimi/gess/RessShowRessource.do?ressourceId=5956**

71. International Labour Organization. (2008). *ILO Declaration on Social Justice for a Fair Globalization*. Geneva. Retrieved from **http://www.ilo.org/global/standards/WCMS_099766/lang--en/index.htm**

72. *International Labour Organization. (2009). *Protect the future: Maternity, paternity and work*. Geneva. Retrieved from **http://www.ilo.org/wcmsp5/groups/public/@dgreports/@gender/documents/publication/wcms_106262.pdf**

73. *International Labour Organization. (2010). *World social security report*. Geneva. Retrieved from **http://www.ilo.org/gimi/gess/ShowTheme.do?tid=1985&lang=EN**

74. Japan International Cooperation Agency. (2011). *Thematic Guidelines on Poverty Reduction*. Tokyo. Retrieved from **http://www.jica.go.jp/english/our_work/thematic_issues/poverty/pdf/guideline.pdf**

75. Japan International Cooperation Agency. (2010). *JICA' s Operation in Health Sector - Present and Future -*. Tokyo. Retrieved from **http://www.jica.go.jp/english/our_work/thematic_issues/health/pdf/position_paper.pdf**

76. Japan International Cooperation Agency. (2009). *Social Security (Health Care/Pension/Social Welfare)*. Tokyo. Retrieved from **http://www.jica.go.jp/english/our_work/thematic_issues/social/pdf/guideline_social.pdf**

77. *Joint United Nations Programme on HIV/AIDS. (2010). *Getting to Zero: UNAIDS 2011-2015 strategy*. Geneva. Retrieved from **http://www.unaids.org/en/media/unaids/contentassets/documents/unaidspublication/2010/JC2034_UNAIDS_Strategy_en.pdf**

78. Joint United Nations Programme on HIV/AIDS. (2010). *Agenda for Accelerated Country Action for Women, Girls, Gender Equality and HIV. Operational plan for the UNAIDS action framework: addressing women, girls, gender equality and HIV*. Geneva. Retrieved from **http://www.unfpa.org/webdav/site/global/shared/documents/publications/2010/agenda_for_accelerated_country_action_en.pdf**

79. *Joint United Nations Programme on HIV/AIDS. (2011). *Countdown to zero: Global plan towards the elimination of new HIV infections among children by 2015 and keeping their mothers alive*. Geneva. Retrieved from **http://www.unaids.org/en/media/unaids/contentassets/documents/unaidspublication/2011/20110609_JC2137_Global-Plan-Elimination-HIV-Children_en.pdf**

80. *MDG Africa Steering Group. (2008). *Achieving the Millennium Development Goals in Africa*. Retrieved from **http://www.who.int/pmnch/events/2008/mdgsteeringgrouprecommendations.pdf**

81. *Médecins du Monde France. (2008). *L'accès gratuit aux soins de santé primaire : une stratégie payante. Appel au G8*. Paris. Retrieved from **http://www.medecinsdumonde.org/Presse/Dossiers-de-presse/A-l-International/L-acces-gratuit-aux-soins-de-sante-primaire-une-strategie-payante-Appel-au-G8**

82. *Médecins sans frontières Belgique. (2008). *No cash, no care: How "user fees" endanger health. An MSF briefing paper on financial barriers to healthcare*. Brussels. Retrieved from **http://www.msf.org/source/access/2008/NocashNocareMSFapril2008.pdf**

83. *Médecins sans frontières Belgique. (2008). *Full prescription: better malaria treatment for more people, MSF's experience*. Brussels. Retrieved from **http://www.msf.org/source/medical/malaria/2008/MSF_malaria_2008.pdf**

84. *Merlin. (2007). *Merlin's role in health financing*. London. Retrieved from **http://www.merlin.org.uk/userfiles/MerlinHealthFinance.pdf** **(not available)**

85. *Merlin, Durrani, N., & Campbell, F. (2010). *Merlin response to the UK Malaria Review*. London. Retrieved from **http://www.merlin.org.uk/news-media/publications** **(not available)**

86. *Merlin, & Keith, R. (2009). *All mothers matter: Investing in health workers to save lives in fragile states*. London. Retrieved from **http://www.healthandfragilestates.org/index.php?option=com_docman&task=doc_download&gid=51&&Itemid=38**

87. *Merlin, & Neal, S. (2010). *Merlin response to Choice for Women: Wanted Pregnancies, Safe Births.* London. Retrieved from **http://www.merlin.org.uk/news-media/publications** **(not available)**

88. Ministère des affaires étrangères et européennes. (2010). *France and the MDGs*. Paris. Retrieved from **http://www.diplomatie.gouv.fr/fr/IMG/pdf/France_and_the_MGDs_2010_en.pdf**

89. Ministère des affaires étrangères et européennes. (2011). *Development Cooperation: a French vision. Strategy 2011. Framework Document*. 2011. Retrieved from **http://www.diplomatie.gouv.fr/fr/IMG/pdf/Doc_Cadre_ANG_2011.pdf**

90. *Ministry of Foreign Affairs of Denmark. (2009). *Health and Development: A Guidance Note to Danish Development Assistance to Health*. Copenhagen. Retrieved from **http://www.enrecahealth.dk/news/GNH_Final_18_NOV_2009_Final.pdf/**

91. Ministry of Foreign Affairs of Sweden. (2007). *Strategy for multilateral development cooperation.* Stockholm. **Retrieved from http://www.government.se/sb/d/3365/a/97680**

92. *Ministry of Foreign Affairs of Sweden. (2008). *Sweden and Africa - a policy to address common challenges and opportunities*. Stockholm. Retrieved from **http://www.government.se/sb/d/9807/a/105300**

93. Ministry of Foreign Affairs of Sweden. (2010). *Guidelines for cooperation strategies*. Stockholm. Retrieved from **http://www.sida.se/Global/About%20Sida/Så%20styrs%20vi/Guidelines%20for%20Cooperation%20Strategies.pdf**

94. Norwegian Agency for Development Cooperation. (2009). *Principles for Norad's Support to Civil Society in the South*. Oslo. Retrieved from **http://www.norad.no/en/_attachment/127633/binary/48700?download=true**

95. Organisation for Economic Co-operation and Development. (2006). *Promoting Pro-Poor Growth: Key Policy Messages*. Paris. Retrieved from **http://www.oecd.org/dataoecd/0/61/37852580.pdf**

96. *Organisation for Economic Co-operation and Development. (2010). *Investing in women and girls: The breakthrough strategy for achieving all the MDGs*. Helsinki. Retrieved from **http://www.oecd.org/social/genderequalityanddevelopment/45704694.pdf**

97. Organisation Ouest Africaine de la Santé. (2008). *Plan Stratégique 2009-2013*. Bobo-Dioulasso. Retrieved from **http://www.wahooas.org/IMG/pdf/PS-2009-2013-FR_q_web.pdf**

98. *Oxfam International. (2008). *If Not Now, When?* Oxford. Retrieved from **http://www.oxfam.org/en/policy/if-not-now-when**

99. *Oxfam International. (2009). *Blind Optimism: Challenging the myths about private health care in poor countries*. Oxford. Retrieved from **http://www.oxfam.org/sites/www.oxfam.org/files/bp125-blind-optimism-0902.pdf**

100. *Oxfam International. (2006). *In the Public Interest: Health, Education, and Water and Sanitation for All*. Oxford. Retrieved from **http://www.oxfam.ca/sites/default/files/in-the-public-interest-health-education-and-water-and-sanitation-for-all.pdf**

101. *People's Health Movement. (2009). *People's Charter for Health*. Cape Town. Retrieved from **www.phmovement.org/files/phm-pch-english.pdf**

102. People's Health Movement. (2005). *The Cuenca Declaration*. Cuenca. Retrieved from **http://www.phmovement.org/cms/files/english%20version.pdf**

103. *Providing for Health (P4H). (2008). Initiative on Social Health Protection. Retrieved from **www.ilo.org/gimi/gess/RessFileDownload.do?ressourceId=10111**

104. *Save the Children. (2010). *A fair chance at life: why equity matters for child mortality*. London. Retrieved from **http://www.savethechildren.org.uk/sites/default/files/docs/A_Fair_Chance_at_Life_1.pdf**

105. *Save the Children UK. (2005). *An Unnecessary Evil? User fees for healthcare in low-income countries* (Vol. 44). London. Retrieved from **http://www.savethechildren.org.uk/sites/default/files/docs/An_Unnecessary_Evil_1.pdf**

106. *Save the Children UK. (2010). *Child survival: what the UK government must do in 2010*. London. Retrieved from **http://www.savethechildren.org.uk/sites/default/files/docs/child_survival_UK_policy_brief_1.pdf**

107. *Save the Children UK. (2011). *No child born to die: closing the gaps*. London. Retrieved from **http://www.savethechildren.org.uk/sites/default/files/docs/No_Child_Born_to_Die_low_res_1.pdf**

108. *Save the Children UK. (2009). *Health user fees – the case against*. London. Retrieved from **http://www.savethechildren.org.uk/sites/default/files/docs/User_fees_FE_edit_1.pdf**

109. *Save the Children UK. (2008). *Saving children's lives: Why equity matters*. London. Retrieved from **http://www.savethechildren.org.uk/sites/default/files/docs/saving-childrens-lives_1.pdf**

110. *Save the Children UK. (2008). *Helping Children Survive: Supporting poor families to overcome barriers to maternal, newborn and child health services*. London. Retrieved from **http://www.savethechildren.org.uk/sites/default/files/docs/Helping_Children_Survive_1.pdf**

111. *Save the Children UK. (2010). *Save the Children's calls to the G8 and G20 in 2010*. London. Retrieved from **http://www.savethechildren.org.uk/sites/default/files/docs/G8_and_G20_calls_1.pdf**

112. *Save the Children UK. (2005). *Killer bills: Make Child Poverty History - Abolish user fees*. London: Save The Children. Retrieved from **http://images.savethechildren.it/IT/f/img_pubblicazioni/img100_b.pdf**

113. *Save the Children UK. (2005). *The Cost of Coping with Illness*. London. Retrieved from **http://www.savethechildren.org.uk/sites/default/files/docs/The_Cost_of_Coping_with_Illness_1.pdf**

114. *Save the Children UK. (2010). *The interdependence of maternal, newborn and child health*. London. Retrieved from **http://www.savethechildren.org.uk/sites/default/files/docs/Interdependance_of_maternal_and_child_health_1.pdf**

115. *Save the Children UK, Keith, R., & Shackleton, P. (2006). *Paying with their Lives: The cost of illness for children in Africa* (p. 24). London. Retrieved from **http://www.savethechildren.org.uk/sites/default/files/docs/paying_with_their_lives%281%29_1.pdf**

116. *Sixty-fourth World Health Assembly. (2011). *Sustainable health financing structures and universal coverage*. New York. Retrieved from **http://apps.who.int/gb/ebwha/pdf_files/WHA64/A64_R9-en.pdf**

117. *Task Force on Global Action for Health System Strengthening. (2009). *Global Action for Health System Strengthening*. Tokyo. Retrieved from **http://www.jcie.org/researchpdfs/takemi/full.pdf**

118. *Taskforce on Innovative International Financing for Health Systems. (2009). *More money for health, and more health for the money*. Geneva. Retrieved from **http://www.internationalhealthpartnership.net/fileadmin/uploads/ihp/Documents/Results___Evidence/HAE__results___lessons/Taskforce_report_EN.2009.pdf**

119. The Global Call to Action Against Poverty. (2011). *The Global Call To Action Against Poverty: Strategic Plan 2011-2014*. Retrieved from **http://whiteband.org/sites/default/files/GCAP StrategicPlan 2011to14 FINAL.pdf**

120. *The Global Campaign for the Health Millennium Development Goals. (2009). *Leading by Example – Protecting the most vulnerable during the economic crisis*. Oslo. Retrieved from **http://www.norad.no/en/thematic-areas/global-health/maternal-child-and-womens-health/health-mdgs-reports/_attachment/385329?_download=true&_ts=1333b72a57d**

121. *The Global Campaign for the Health Millennium Development Goals. (2010). *Putting the Global Strategy for Women's and Children's Health into action*. Oslo. Retrieved from **http://www.norad.no/en/thematic-areas/global-health/maternal-child-and-womens-health/health-mdgs-reports/_attachment/380929?_download=true&_ts=13104abc70a**

122. *The Global Coalition on Women and AIDS. (2006). *Keeping the Promise: An Agenda for Action on Women and AIDS*. Geneva. Retrieved from

http://www.unfpa.org/webdav/site/global/shared/documents/publications/2006/keeping_promise.pdf

123. The Netherlands Ministry of Foreign Affairs. (2009). *Choices and opportunities*. The Hague. Retrieved from **http://www1.minbuza.nl/dsresource?objectid=buzabeheer:79640&type=org (not available)**

124. *The World Bank. (2010). *The World Bank's Reproductive Health Action Plan 2010-2015*. New York. Retrieved from **http://siteresources.worldbank.org/INTPRH/Resources/376374-1261312056980/RHActionPlanFinalMay112010.pdf**

125. The World Bank. (2005). *Meeting the Challenge of Africa's Development: A World Bank Group Action Plan*. Washington, D.C. Retrieved from **http://siteresources.worldbank.org/INTAFRICA/Resources/aap_final.pdf**

126. *The World Bank. (2007). *Healthy Development: The World Bank Strategy for Health, Nutrition and Population Results*. Washington, D.C. Retrieved from **http://siteresources.worldbank.org/HEALTHNUTRITIONANDPOPULATION/Resources/281627-1154048816360/HNPStrategyFINALApril302007.pdf**

127. U.S. Department of State, & U.S. Agency for International Development. (2007). *Strategic Plan Fiscal Years 2007-2012*. Washington, D.C. Retrieved from **http://transition.usaid.gov/performance/stratplan/stratplan_fy07-12.pdf**

128. *UN Millennium Project. (2005). *Preparing National Strategies to Achieve the Millennium Development Goals: A Handbook*. Washington. Retrieved from **http://www.gm.undp.org/Reports/Preparing%20national%20strategies%20to%20achieve%20the%20MDGs.pdf**

129. *UN Millennium Project (Task Force on Child Health and Maternal Health). (2005). *Who's got the power? Transforming health systems for women and children. Achieving the Millennium Development Goals Development*. London. Retrieved from **http://www.unmillenniumproject.org/documents/maternalchild-complete.pdf**

130. United Nations General Assembly. (2011). *Political Declaration on HIV/AIDS: Intensifying our Efforts to Eliminate HIV/AIDS*. New York. Retrieved from **http://www.unaids.org/en/media/unaids/contentassets/documents/document/2011/06/20110610_UN_A-RES-65-277_en.pdf**

131. United Nations General Assembly. (2010). *Keeping the promise: united to achieve the Millennium Development Goals*. New York. Retrieved from **http://www.un.org/en/mdg/summit2010/pdf/mdg outcome document.pdf**

132. United Nations Population Fund. (2005). *Reducing Poverty and Achieving The Millennium Development Goals: Arguments for investing in reproductive health and rights. Reference Notes on Population and Poverty Reduction*. Paris. Retrieved from **http://www.unfpa.org/webdav/site/global/shared/documents/publications/2005/reducing poverty_mdg.pdf**

133. USAID. (2010). *Celebrate, Innovate & Sustain. Toward 2015 and Beyond. The United States' Strategy for Meeting the Millennium Development Goals*. Washington. Retrieved from **http://www.usaid.gov/our_work/mdg/USMDGStrategy.pdf**

134. USAID. (2008). *Working toward the goal of reducing maternal and child mortality: USAID programming and response to FY08 appropriations*. Washington, D.C. Retrieved from **http://pdf.usaid.gov/pdf_docs/PDACL707.pdf**

135. *World Health Organization. (2010). *Health Systems Financing: The path to universal coverage. The World Health Report*. Geneva. Retrieved from **http://whqlibdoc.who.int/whr/2010/9789241564021_eng.pdf**

136. *World Health Organization. (2008). *The world health report 2008: primary health care now more than ever*. Geneva. Retrieved from **http://www.who.int/whr/2008/whr08_en.pdf**

137. *World Vision. (2009). *Who's counting? 9.2 million children - the cost of inaction on child health*. Monrovia. Retrieved from **http://www.wvi.org/wvi/wviweb.nsf/11FBDA878493AC7A882574CD0074E7FD/$file/Who-s-Counting-report.pdf**

138. *World Vision. (2009). *Child health now: together we can end preventable deaths*. Monrovia. Retrieved from **http://www.who.int/pmnch/topics/child/child_health_now.pdf**

139. *World Vision. (2009). *Why the G8 matters to children: World Vision's policy calls to the 2009 G8 Summit*. Monrovia. Retrieved from **http://www.globalempowerment.org/PolicyAdvocacy/pahome2.5.nsf/alleports/073DCFF71ADD29258825758A0020E2EC/$file/G8 2009 BP_WEB.pdf**

140. *World Vision. (2008). *Last chance for the world to live up to its promises? Why decisive action is needed now on child health and the MDGs*. Monrovia. Retrieved from **http://www.wvi.org/wvi/wviweb.nsf/A5855068762B65C28825784D0077B1A4/$file/LastChance_WEB.pdf**

* Documents included in thematic analysis.
