# Supplementary material for: Global health actors no longer in favor of user fees: a documentary study
Source: Global Health. 2013 Jul 26;9:29. doi: 10.1186/1744-8603-9-29 (PMC3750575; doi:10.1186/1744-8603-9-29)
Supplement: Additional file 2 — Characteristics of the positive or nuanced stance of GHAs. [file 1744-8603-9-29-S2.pdf]

CHARACTERISTICS OF THE POSITIVE OR NUANCED STANCE OF GHAs

GHAs*	Suggested measure	Targeted populations	Targeted services	Combination or other
International governmental organizations (IGOs) *(n=8)*				
**African Union** (n=2)	Other financing scheme [1]	-	-	-
	User fee exemption / abolition + other financing scheme [2]	-	-	-
**CEU** (n=3)	(Universal) free access / free care [3]	-	HIV/AIDS prevention, treatment and care	-
	Other financing scheme [4]	-	-	Essential health services for vulnerable populations
	(Universal) free access / free care [5]	-	Basic health care	-
**ECHO** (n=1)	User fee exemption / abolition [6]	-	-	During humanitarian emergencies
**European Commission** (n=1)	(Universal) free access / free care [7]	-	-	HIV/AIDS prevention, treatment and care for pregnant women
**International Labour Office** (n=5)	Other financing scheme [8]	Women	-	-
	Other financing scheme [9-11]	-	-	-
	(Universal) free access / free care [12]	-	-	Pre-natal and post-natal care, childbirth, hospitalizations for mothers
**World Health Assembly** (n=1)	Other financing scheme [13]	-	-	-
**The World Bank** (n=2)	User fee exemption / abolition [14]	Vulnerable / poor populations	-	-
	(Universal) free access / free care [15]	Mothers and children	-	-
**WHO** (n=2)	Other financing scheme [16, 17]	-	Primary health care	-
Government agencies (GAs) *(n=3)*				
**Denmark** (n=1)	Other financing scheme [18]	-	-	Primary health care for children and pregnant women
**Germany** (n=1)	(Universal) free access / free care [19]	-	HIV/AIDS prevention, treatment and care	-

GHAs*	Suggested measure	Targeted populations	Targeted services	Combination or other
**United Kingdom** (n=3)	User fee exemption / abolition [20]	Women + Children	-	-
	User fee exemption / abolition [21]	-	Basic health care	-
	User fee exemption / abolition [22]	-	Essential health care	-
International non-governmental organizations (INGOs) *(n=6)*				
**MDM** (n=1)	User fee exemption / abolition [23]	-	Primary health care	-
**Merlin** (n=3)	Other financing scheme [24]	-	Basic health care	-
	Other financing scheme [25]	Mothers	-	-
	Other financing scheme [26]	Mothers and children	-	-
**MSF** (n=2)	(Universal) free access / free care [27]	-	Malaria diagnosis and treatment	-
	(Universal) free access / free care [28]	-	-	-
**Oxfam** (n=3)	User fee exemption / abolition [29, 30]	-	-	-
	(Universal) free access / free care [31]	-	Basic health care	-
**Save the Children** (n=10)	Other financing scheme [32]	Mothers and children	-	-
	(Universal) free access / free care [33, 34]	Mothers and children	-	-
	(Universal) free access / free care [35, 36]	Children	-	-
	User fee exemption / abolition [37]	Children	-	-
	User fee exemption / abolition [38-40]	-	-	-
	(Universal) free access / free care [41]	-	Essential health care	-
**World Vision** (n=2)	(Universal) free access / free care [42]	Mothers and children	-	-
	(Universal) free access / free care [43]	-	-	Minimum package of healthcare for children
Networks and working groups (NW/WGs) *(n=14)*				
**Action for Global Health** (n=5)	User fee exemption / abolition [44, 45]	Women + Vulnerable / poor populations	-	-

GHAs*	Suggested measure	Targeted populations	Targeted services	Combination or other
	(Universal) free access / free care [46]	-	-	Basic healthcare for children
	(Universal) free access / free care [47]	Vulnerable / poor populations	Primary health care	-
	(Universal) free access / free care [48]	-	-	-
**AMCP-F** (n=1)	User fee exemption / abolition + other financing scheme [49]	-	Basic healthcare + Long-term illness	-
**Commission for Africa** (n=2)	User fee exemption / abolition [50]	-	Basic healthcare	-
	User fee exemption / abolition [51]	-	-	-
**Coordination Sud** (n=2)	User fee exemption / abolition [52]	Mothers and children	-	-
	User fee exemption / abolition [53]	Vulnerable / poor populations	-	-
**Countdown to 2015** (n=1)	(Universal) free access / free care + other financing scheme [54]	Mothers and children	-	-
**G8** (n=2)	(Universal) free access / free care + other financing scheme [55]	-	-	Basic healthcare for children
	(Universal) free access / free care [56]	Mothers and children	-	-
**GHW** (n=1)	User fee exemption / abolition [57]	-	Essential health care	-
**UNAIDS** (n=1)	User fee exemption / abolition [58]	Mothers	-	HIV prevention, treatment and care for women
**MDG Africa Steering Group** (n=1)	User fee exemption / abolition [59]	-	Essential health care	-
**PHM** (n=1)	(Universal) free access / free care [60]	-	Primary health care	-
**Takemi WG** (n=1)	User fee exemption / abolition [61]	-	-	-
**TF on IIFHS** (n=1)	(Universal) free access / free care [62]	-	-	-
**Global Campaign for Health MDGs** (n=1)	(Universal) free access / free care [63]	Vulnerable / poor populations	-	-
**UN Millennium Project** (n=2)	User fee exemption / abolition [64, 65]	-	Basic healthcare	-

**The list of abbreviations is available in the article.*

References

1. African Union: *The new partnership for Africa's development (NEPAD): health strategy*. Addis Ababa; 2007.

2. African Union: *Africa Health Strategy: 2007-2015*. Addis Ababa; 2007.

3. Council of the European Union: *The EU as a global partner for pro-poor and pro-growth development: EU Agenda for Action on MDGs*. Brussels; 2008.

4. Council of the European Union: *Council conclusions on the EU role in Global Health*. Brussels; 2010.

5. Council of the European Union: *Council Conclusions on the promotion and protection of the rights of the child in the European Union's external action - the development and humanitarian dimensions*. Brussels; 2008.

6. ECHO: *DG ECHO position paper on user fees for primary health services in humanitarian crises*. Brussels; 2009.

7. Commission of the European Communities: *A European Programme for Action to Confront HIV/AIDS, Malaria and Tuberculosis through External Action (2007-2011)*. Brussel; 2005.

8. International Labour Organization: *Safe maternity and the world of work*. Geneva; 2007.

9. International Labour Organization: *Social health protection: An ILO strategy towards universal access to health care (a consultation)*. Geneva; 2007.

10. International Labour Office: *Extending social security to all: A guide through challenges and options*. Geneva; 2010.

11. International Labour Organization: *World social security report*. Geneva; 2010.

12. International Labour Office: *Achieving MDG 5 through decent work*. Geneva; 2010.

13. Sixty-fourth World Health Assembly: *Sustainable health financing structures and universal coverage*. New York; 2011.

14. The World Bank: *Healthy development: The World Bank strategy for health, nutrition and population results*. Washington; 2007.

15. The World Bank: *The World Bank's reproductive health action plan 2010-2015*. Washington; 2010.

16. World Health Organization: *The world health report 2008: Primary health care now more than ever*. Geneva; 2008.

17. World Health Organization: *Health systems financing: The path to universal coverage. The World Health Report*. Geneva; 2010.

18. Ministry of Foreign Affairs of Denmark: *Health and development: A guidance note to Danish development assistance to health*. Copenhagen; 2009.

19. Federal Ministry for Economic Cooperation and Development: *Promoting Health - Fighting HIV / AIDS*. Berlin; 2007.

20. Department for International Development: *Eliminating World Poverty: Building Our Common Future*. London; 2009.

21. Department for International Development: *Eliminating world poverty. Making governance work for the poor. A white paper on international development*. London; 2006.

22. Department for International Development: *Working together for better health.* London; 2007.

23. Médecins du Monde: *L'accès gratuit aux soins de santé primaire : Une stratégie payante. Appel au G8.* Paris; 2008.

24. Merlin: *Merlin's role in health financing*. London; 2007.

25. Merlin: *All mothers matter. Investing in health sector to save lives in fragile states*. London; 2009.

26. Merlin: *Merlin response to Choice for Women: Wanted pregnancies, safe births*. London; 2010.

27. Médecins Sans Frontières: *Full prescription. Better malaria treatment for more people, MSF's experience.* Brussels; 2008.

28. Médecins Sans Frontières: *No cash, no care: How "user fees" endanger health. An MSF briefing paper on financial barriers to healthcare.* Brussels; 2008.

29. Oxfam International: *In the public interest: Health, education, and water and sanitation for all*. Oxford; 2006.

30. Oxfam International: *Blind optimism: Challenging the myths about private health care in poor countries*. Oxford; 2009.

31. Oxfam International: *If not now, when?* Oxford; 2008.

32. Save the Children UK: *The interdependence of maternal, newborn and child health*. London; 2010.

33. Save the Children UK: *Save the Children's calls to the G8 and G20 in 2010*. London; 2010.

34. Save the Children UK: *Child survival: What the UK government must do in 2010*. London; 2010.

35. Save the Children UK: *No child born to die: Closing the gaps*. London; 2011.

36. Save the Children UK: *Killer bills: Make child poverty history - abolish user fees*. London; 2005.

37. Save the Children UK: *Saving children's lives: Why equity matters*. London; 2008.

38. Save the Children UK: *Health user fees - The case against*. London; 2009.

39. Save the Children UK: *The cost of coping with illness*. London; 2005.

40. Save the Children UK: *Paying with their live: The cost of illness for children in Africa*. London; 2006.

41. Save the Children UK: *An unnecessary evil? User fees for healthcare in low-income countries*. London; 2005.

42. World Vision: *Who's counting? 9.2 million children – the cost of inaction on child health*. Monrovia; 2009.

43. World Vision: *Child health now: Together we can end preventable deaths*. Monrovia; 2009.

44. Action for Global Health: *Achieving the health MDGs by 2015: What the EU needs to do!* Brussels; 2010.

45. Action for Global Health: *Action for Global Health recommendations for the Council Conclusions on the European Commission's Communication on the EU Role in Global Health*. Brussels; 2010.

46. Action for Global Health: *Improving the health of children across the world needs to be made a priority by governments*. Strasbourg; 2010.

47. Action for Global Health: *2010 reality check: Time is running out to meet the health MDGs*. Brussels; 2010.

48. Action for Global Health: *Dear member of the Development Cooperation Working Group*. Brussels; 2010.

49. Action Mondiale Contre la Pauvreté - France: *Health for development: Challenges and responsibilities: At the midway point in the Millennium development goals, where do we stand on health? The French civil society recommendations*. Paris; 2008.

50. Commission for Africa: *Our common interest: report of the Commission for Africa*. London; 2005.

51. Commission for Africa: *Still our common interest: commission for Africa report 2010*. London; 2010.

52. Coordination Sud: *Proposition des ONG françaises*. Paris; 2010.

53. Coordination Sud: *Préparation du G8 de Hokkaido, Toyako, Japon 7–9 Juillet 2008. Propositions des ONG françaises.* Paris; 2008.

54. Countdown to 2015: *Countdown to 2015 decade report (2000-2010): Taking stock of maternal, newborn and child survival*. Geneva; 2010.

55. G8: *G8 Hokkaido Toyako Summit Leaders Declaration*. Toyako; 2008.

56. G8: *Responsible leadership for a sustainable future*. L'Aquila; 2009.

57. Global Health Watch: *Global Health Watch 2005-2006: An alternative world health report*. London; 2006.

58. Joint United Nations Programme on HIV AIDS: *Countdown to zero: Global plan towards the elimination of new HIV infections among children by 2015 and keeping their mothers alive*. Geneva; 2011.

59. MDG Africa Steering Group: *Achieving the Millennium Development Goals in Africa*. New York; 2008.

60. People's Health Movement: *People's Charter for Health*. Cape Town; 2009.

61. Task Force on Global Action for Health System Strengthening: *Global action for health system strengthening: Policy recommendations to the G8.* Tokyo; 2009.

62. Taskforce on Innovative International Financing for Health Systems: *More money for health, and more health for the money*. Geneva; 2009.

63. The Global Campaign for the Health Millennium Development Goals: *Leading by example – Protecting the most vulnerable during the economic crisis*. Oslo; 2009.

64. UN Millennium Project: *Who's got the power? Transforming health systems for women and children. Achieving the Millennium Development Goals*. London; 2005.

65. UN Millennium Project: *Preparing national strategies to achieve the Millennium Development Goals: A handbook*. Washington; 2005.
